# Fatty acids role in multiple sclerosis as “metabokines”

**DOI:** 10.1186/s12974-022-02502-1

**Published:** 2022-06-17

**Authors:** Haojun Yu, Shuwei Bai, Yong Hao, Yangtai Guan

**Affiliations:** Department of Neurology, Renji Hospital, Shanghai Jiao Tong University School of Medicine, 160 Pujian Road, Pudong, Shanghai, 200127 China

**Keywords:** Fatty acid metabolism, Multiple sclerosis, Immune, Neurodegeneration

## Abstract

Multiple sclerosis (MS), as an autoimmune neurological disease with both genetic and environmental contribution, still lacks effective treatment options among progressive patients, highlighting the need to re-evaluate disease innate properties in search for novel therapeutic targets. Fatty acids (FA) and MS bear an interesting intimate connection. FA and FA metabolism are highly associated with autoimmunity, as the diet-derived circulatory and tissue-resident FAs level and composition can modulate immune cells polarization, differentiation and function, suggesting their broad regulatory role as “metabokines”. In addition, FAs are indeed protective factors for blood–brain barrier integrity, crucial contributors of central nervous system (CNS) chronic inflammation and progressive degeneration, as well as important materials for remyelination. The remaining area of ambiguity requires further exploration into this arena to validate the existed phenomenon, develop novel therapies, and confirm the safety and efficacy of therapeutic intervention targeting FA metabolism.

## Background

Multiple sclerosis (MS), the chronic demyelinating neuroautoimmune disease, is a prevalent cause of disability among the youth, unfortunately still uncurable [1]. Although early-stage MS patients usually experience a partial recovery after each attack, a consistent progressive course will eventually take over, featuring central nervous system (CNS) degeneration and gradually aggravated disability [2, 3], posing challenges for effective treatment [4, 5]. The underlying cause of MS is considered to be a combination of genetic susceptibility [6], lifestyle, and environmental factors [4, 7, 8]. Among those factors, the interest given to fatty acid (FA) in the discovery of MS etiology and therapeutics can be traced back to the 1950s [9, 10], when evidence from epidemiology and autopsy supported a lack of polyunsaturated FAs (PUFAs) consumption [11, 12], and an inadequacy of CNS FA elongation and desaturation [13, 14] among MS patients. Disappointingly, several randomized controlled trials (RCTs) conducted between the 1980s to 2010s have shown no clear benefits of introducing PUFAs into MS therapy [15–18], despite the numerous positive evidence from in vitro and animal studies. It is not until the 2010s when researchers have discovered a coordinating role of diet and gut microbiome in affecting autoimmune disease severity, where FAs featured [19], which partially explains how an unhealthy lifestyle serves as a predisposing factor of MS occurrence. More importantly, the mysterious microbiota-derived metabolites, namely, short-chain FAs (SCFAs), have further established an extensive regulatory role of biological events [20], and have recently achieved optimistic results as an add-on therapy of MS treatment [21], therefore, drawing back attention to the perspective of FAs-targeted therapy.

Plentiful work has been endeavored to clarify this issue, either focusing on the general role of FAs in physiological or pathological circumstances [22–25] or on the beneficial effect of dietary FAs supplementation with data from clinical trials [26]. Even though, the understanding of FAs’ role in MS is still insufficient. First, considering the previous molecular-oriented view, there is a lack of disease-oriented interpretations with in-depth information, which could be more easily accessible for clinicians and researchers. Second, recent research advances call for more intensive attention on their functions in the neuroinflammatory aspect beyond traditional metabolic roles [27]. Herein, this review aims to provide proof for the intimate connection between FAs and MS with an integrated perspective from molecular to systematic levels. We point out FAs’ function as “metabokines” to recapitulate their role as the crucial environmental factor participating in MS pathogenesis, bridging the gap between metabolism and neuroinflammation while aiding further exploration into their actual therapeutic value.

## A brief introduction to FA metabolism

**Fig. 1:**
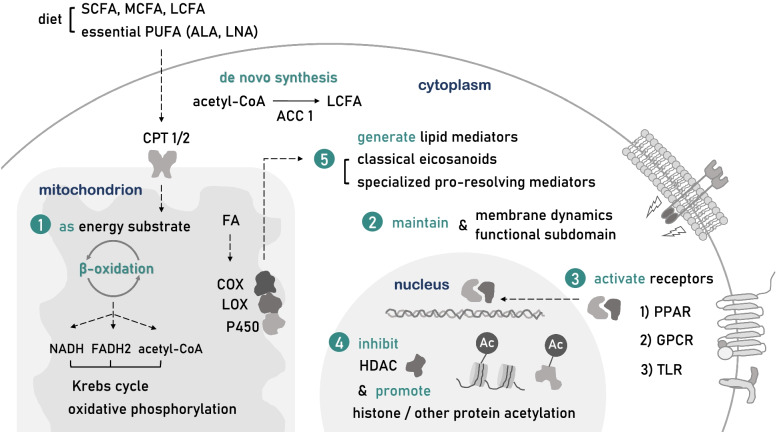
FAs as metabokines at a molecular level. SCFAs, MCFAs, LCFAs, and essential PUFAs are mostly obtained from diet, whereas LCFAs can also be synthesized de novo which requires the participation of rate-limiting enzymes, such as acetyl-CoA carboxylases1 (ACC1). The FA β-oxidation process starts with the translocation into mitochondrion assisted by critical transporter carnitine palmitoyl-transferase 1/2 (CPT1/2). The extensive regulatory role of FAs derives from generally five ways of action. (1) FAs are energy substrates that produce NADH, acetyl-CoA, and FADH_2_ to support the Krebs cycle and oxidative phosphorylation in the mitochondrion. (2) FAs are responsible for membrane dynamics and through the alteration of FA level and composition regulate local membrane biological functions. (3) FAs activate various membrane and nuclear receptors including GPCRs, TLRs, PPARs, affecting downstream signaling pathways. (4) FAs, especially short-chain FAs, are potent histone deacetylase (HDAC) inhibitors capable of regulating histone or non-histone acetylation to modulate the expression and stability of transcripts and proteins. (5) FAs through metabolism generate downstream lipid mediators, widely participating in the maintenance and resolution of chronic inflammation

FA, structurally a hydrocarbon chain with a terminal hydrophilic carboxylate group, can be classified according to the length of the carbon chain and the property of double bonds [28, 29] (Table 1). Biological SCFAs are end products from intestinal bacteria fermentation of a high fiber diet, whereas MCFAs are saturated or unsaturated FAs found at high concentrations in food, such as dietary milk fat and coconut oil [30]. Apart from dietary intake, the de novo synthesis of FAs requires the participation of various rate-limiting enzymes including acetyl-CoA carboxylases (ACCs), fatty acid synthases (FASNs), and elongases (Fig. 1). Due to the lack of desaturases, initial PUFAs are required from the diet to provide basic materials for the subsequent synthesis of other PUFAs. Template PUFAs, namely, α‑linolenic acid (ALA) and omega‑6 linoleic acid (LNA) are so-called essential FAs [23, 31, 32].

**Table 1:** Fatty acid (FA) classification and nomenclature

SCFA^A^	C1	C2	C3	C4	C5	Synthetic
	Formic acid	Acetic acid	Propionic acid	Butyric acid	Valeric acid	Valproic acid (VPA)

MCFA	C6	C8	C10	C12
	Caproic acid	Caprylic acid	Capric acid	Lauric acid

LCFA	C16	C18	C20	C22
SFA^B^	Palmitic acid C16:0	Stearic acid C18:0	Arachidic acid C20:0	Behenic acid C22:0
MUFA	Palmitoleic acid C16:1	Oleic acid C18:1	Gondoic acid C20:1	Erucic acid C22:1
Omega-6 PUFA		Linoleic acid C18:2 (LNA)	Dihomo-γ-linoleic acid C20:3	Docosapenaenoic acid C22:5
		γ-linoleic acid C18:3	Arachidonic acid C20:4 (ARA)	
Omega-3 PUFA		α-linoleic acid C18:3 (ALA)	Eicosatetranoic acid C20:4 (EPA)	Docosahexanoic acid C22:6 (DHA)
		Stearidonic acid C18:4	Dicosapentaenioc acid C20:5	

A: FAs are categorized according to the length of the carbon chain, and the number, position of double bonds. Short-chain fatty acids (SCFAs) refer to those that contain 1–5 carbon atoms, while medium-chain fatty acids (MCFAs) are those of 6–12, and long-chain fatty acids (LCFAs) of 14–22, very-long-chain fatty acids (VLCFA) of > 24. B: The number of double bonds decides whether they are saturated (SFAs), or mono-/poly-unsaturated fatty acids (M/PUFAs)

*LCFA* long-chain fatty acid, *MCFA* medium-chain fatty acid, *MUFA* mono-unsaturated fatty acid, *PUFA* poly-unsaturated fatty acid, *SCFA* short-chain fatty acid, *SFA* saturated fatty acid

FAs can easily enter CNS. MCFAs and SCFAs cross the blood–brain barrier (BBB) mainly through passive diffusion in the form of free fatty acids [33, 34], while PUFAs also rely on transcytosis [28, 35]. After entering the CNS, most of the PUFAs are esterified into the cell membrane, practicing biological functions upon dissociation [28]. The highly excitable CNS contains abundant FAs. Saturated FAs (SFAs) and mono-unsaturated FAs (MUFAs) are usually synthesized de novo. However, omega-3/6 PUFAs also require peripheral blood replenishment due to low CNS synthesis rate [28]. The stability of CNS membrane lipid composition depends greatly on dietary supply, especially for developing embryos [36].

## Intimate relationship between FAs and MS

The necessity of exploring FA contribution in MS is well-founded. As the major components of oligodendrocyte membrane, it is not surprising that systemic and CNS resident FA level undergo remarkable fluctuation during demyelination, confirmed as a unique MS FA profile [37–40], which is correlated with disease severity [41–43] and activity [44], even for those symptom-free preclinical patients [40]. At the same time, the highly dynamic CNS under remyelination requires abundant peripheral essential FAs as materials for reconstruction [45]. However, it seems arbitrary to merely regard the alteration of MS biofluid or membrane FA composition as a reflection of CNS demyelination process, or it could unveil an FA metabolic state that brings more susceptibility.

In fact, FA metabolism-related genes and proteins are proved to be associated with MS incidence and prognosis. Fatty acid desaturase 2 (*FADS2*) single nucleotide polymorphisms (SNPs) rs174611 and rs174618 are both independent protective factors of MS incidence [46], and a higher level of serum fatty acid-binding protein (FABP4) associates with more severe symptoms evaluated by EDSS score [47]. A novel FABP5/7 inhibitor MF6 is proved to attenuate CNS demyelination in MS animal models by modulating inflammatory cytokines production of astrocytes and microglia, thus supporting oligodendrocytes survival by mitigating mitochondria oxidative stress [48].

Moreover, FAs are critical components of gut–brain axis. The perturbation of gut–brain axis has recently been recognized as a characteristic of MS pathology including altered gut microbiota composition [49] and increased intestinal barrier permeability [50]. On the one hand, fecal metagenomic sequencing revealed a detrimental effect of MS gut microbiota which is majorly attributed to the loss of SCFAs-producing bacteria [19, 51]. Specifically, SCFAs and MCFAs by exercising either protective or damaging effects, mediate the complicated crosstalk between gut microbiota and immune system [52, 53], as will be discussed later [54]. On the other hand, research from other autoimmune diseases indicates that FAs such as butyric acid protects intestinal epithelial cells against environmental stress and preserves intestinal barrier integrity by reducing pro-inflammatory cytokines production while inducing tight junction proteins and mucin expression [51, 55, 56]. Therefore, FAs can be crucial therapeutic targets in restoring gut–brain axis perturbation.

FAs level measurement has several clinical implications in MS. A predictive role of serum butyric acid level is established in evaluating MS intestinal barrier permeability [57]. Similarly, caproic acid level defines Th1 lymphocytes level, while the ratio between butyric acid/caproic acid concentration reflects Treg–Th1 axis balance [57]. Acetic acid plasma levels are reported to correlate EDSS [58]. A prospective cohort that included 53 pregnant MS patients and 21 healthy controls points out a correlation between low serum propionate/acetate ratio during the first trimester of gestation and the risk of relapses during pregnancy and postpartum period [59]. It is also worth noting that the therapeutic effect of dimethyl fumarate (DMF) could be reflected by FA metabolism. Among MS patients receiving DMF treatment, a significant change of plasma SFA and MUFA level can be observed, and is correlated with the drop of lymphocyte counts and CD8^+^T cell compartments, reflecting the effectiveness of immunomodulation [60]. These findings lead us to the feasibility of FAs serological concentration as a simple and rapid biomarker in evaluating disease activity, short-term prognosis and treatment efficacy.

Therefore, the intimate connection between FAs and MS has solid biological and clinical foundation. FAs or FA metabolism could indeed serve as the predisposing factor of MS incidence and progression, constituting a part of metabolic memory which takes shape through long-term epigenetic manipulation [61]. As a proof-of-concept, immune cells, one of the most widely modulated by epigenetics throughout their lifespan [62], are extensively manipulated by FAs and FA metabolism. Nevertheless, FAs or FA metabolism actually bear part of MS CNS regeneration potential. Adequate peripheral FAs replenishment, and rapid oligodendrocytes FAs de novo synthesis are required for efficient remyelination [45]. However, once per-oxidized, FAs are responsible for the spread of oxidative damage [63].

## FAs as “metabokines”

Owing to decades of research, FAs or FA metabolism have established a broad regulative role of biological systems, beyond our traditional understanding. It is worth noting that FAs as metabolites, indeed harbor the ability to manipulate epigenetics and cell signal transduction, etc., reflecting their identity as “metabokines”. As this review aims to illustrate how FAs participate in MS pathogenesis, we prepare to recapitulate their ways of function at a molecular level in advance (Fig. 1).

First, FAs are energy substrates that through β-oxidation generate acetyl-CoA, NADH, and FADH_2_ to support the Krebs cycle and oxidative phosphorylation. β-oxidation is initiated by the activation of FAs, the majority of which are then transported by carnitine palmitoyl-transferase 1/2 (CPT1/2) into the mitochondrial matrix, where the principal process takes place [23]. SCFAs and MCFAs are more effective energy substrates compared to LCFAs, since their absorption into enterocytes is more rapid and their metabolism does not require chaperones or transporters [33]. FAs are also the innate regulator of cellular metabolism, while exogenous FAs manipulate metabolism in a cell type-specific way. As FAs inhibit glycolysis and promote anaerobic glucose metabolism and lipid synthesis in hepatocytes, they promote glycolysis in astrocytes, generating lactate into the extracellular environment to support surrounding cells [33].

Second, LCFAs are responsible for the stability of cytomembrane dynamics and function. FAs provide important materials for phospholipid and sphingolipid biosynthesis [64]. The content and proportion of endogenous or exogenous LCFAs can directly affect the lipid composition of the dynamic phospholipid bilayer, defining its innate properties including fluidity, permeability, and compressibility [65, 66]. Meanwhile, different local FAs composition leads to the formation of structurally and functionally unique lipid raft subdomain [66], facilitating biological processes, such as transmembrane transport, cell signal transduction, etc. [36]. For instance, membrane PUFAs composition can affect the function of glycoprotein receptors floating on lipid rafts. In the research of fat-1 mice, where omega-3 PUFAs undergo spontaneous transformation into omega-6 PUFAs, a reduction of membrane GP130 expression is witnessed in CD4^+^T lymphocytes, accompanied by the difficulty of GP130 homodimerization necessary for downstream signal transduction, which hinders their differentiation into Th17 cells prompted by IL-6 [67].

Third, FAs are ligands for a series of membrane and nuclear receptors, such as (1) G-protein coupled receptors (GPCR), including GPR41/43/109a/84/40/120; (2) Toll-like receptors (TLR), including TLR2/4; as well as (3) peroxisome proliferator-activated receptors (PPARs), including PPAR-α, γ, β/δ. FAs activating GPCRs not only modulates cell metabolism by promoting glucose-induced insulin and GLP1 secretion but also exhibit broad immune- and neuro-regulative effects. LCFAs, by activating immunocyte-distributed GPR40/120, can inhibit their proinflammatory response and induce macrophage M2 polarization, whereas acting on CNS receptors can regulate cognitive impairment, chronic pain, and epilepsy. MCFAs can bind GPR84 expressed on leukocytes and microglia yet their effects are still elusive. In addition, SCFAs activating GPR43 in neutrophils inhibit their chemotaxis or secretion of inflammatory cytokines, while a GPR41 activation on dendritic cells would enhance their induction of Th2 cell response [68]. Interestingly, SCFAs by activating GPR43 can lead to a change of cell membrane potentials accompanying K^+^ efflux and intracellular Ca^2+^i mobilization [69]. SFAs can promote, whereas DHA restrains TLR2/4 downstream proinflammatory signaling pathway in macrophages and neutrophils [70]. FAs acting on nuclear receptors PPAR-α, γ, β/δ would form heterodimers with RXR, which as transcription factors (TF) regulate FA metabolism-related gene expression, promote mitochondrial biogenesis and alleviate oxidative stress, also participate in NF-κB associated inflammation [71–73].

Fourth, SCFAs, by serving as histone deacetylase (HDAC) class I/IIb inhibitor [74], regulate histone acetylation and crotonylation, meddling with epigenetics through post-translational modification (PTM). SCFAs regulate the transcriptional accessibility of the genome, to interfere with related gene expression in a cell-specific, dose and time-dependent manner. Branched-chain SCFA valproic acid (VPA) as HDAC inhibitor acts differently on the proliferation rate and motility of diverged cancer cell lines [75]. Butyrate promotes apoptosis in neutrophils of either activated or inactivated status [76], however, boosts proliferation in Treg cells [77]. Butyrate and propionate regulate B lymphocyte antibody production in a dose-dependent manner, as low dose promotes, but high dose reduces the generation of systemic specific antibodies [78]. 30 min treatment of butyrate in epithelial cells can sensitize TLR, TNF-α-related downstream activation of NF-κB, reduce IL-8 and chemokine (C–C motif) ligand 2 (CCL2) expression [79], while a long-term treatment of more than 24 h promotes cyclin D3 and p21 expression, arresting cells at G1 phase [80]. SCFAs such as butyrate by targeting HDAC1/2/3 alters histone crotonylation level in the small intestine, colon, and putatively brain, thus participating in cell cycle modulation [81]. Furthermore, SCFAs can also regulate the expression level and cytoplasmic translocation of RNA-binding protein (RBP), such as tristetraprolin [82–84] and HuR [85], to affect their interaction with mRNA AU-rich elements (ARE), manipulating the stabilization and degradation of targeted transcripts.

Fifth, FAs generate downstream hormone-like lipid mediators which act immediately on local receptors to modulate the inflammatory response. Omega-3 and omega-6 PUFAs through cyclooxygenase (COX), lipoxygenase (LOX), or cytochrome P450 oxidase would engender either classical eicosanoids including prostaglandins (PG), thromboxanes (TX), or specialized pro-resolving mediators (SPMs), such as resolvins, protectins, maresins, etc. [86]. Among them, classical eicosanoids act to mediate the occurrence and maintenance of chronic inflammation, by promoting the expression of inflammatory factors, enhancing innate immunity response towards pathogen-associated molecular pattern (PAMP), and damage-associated molecular pattern (DAMP), and activating proinflammatory Th subtypes, such as Th1 and Th17 [87]. In contrast, SPMs help in the resolution of inflammatory response and the remodeling of innate homeostasis, by reducing ROS production, introducing the secretion of anti-inflammatory factors, recruiting scavenging macrophages, and regulating T, B lymphocytes functions [88].

## FAs with immunomodulatory properties in MS

MS is widely acknowledged as an autoimmune disease driven by T lymphocytes, particularly autoimmune Th1, Th17 cells, and aided by B lymphocytes and other innate immune cells [5, 89]. FAs and FA metabolism have demonstrated a broad immunomodulatory capacity as will be discussed as follows.

### FA metabolism and intracellular FA balance modulate T lymphocytes function

**Fig. 2:**
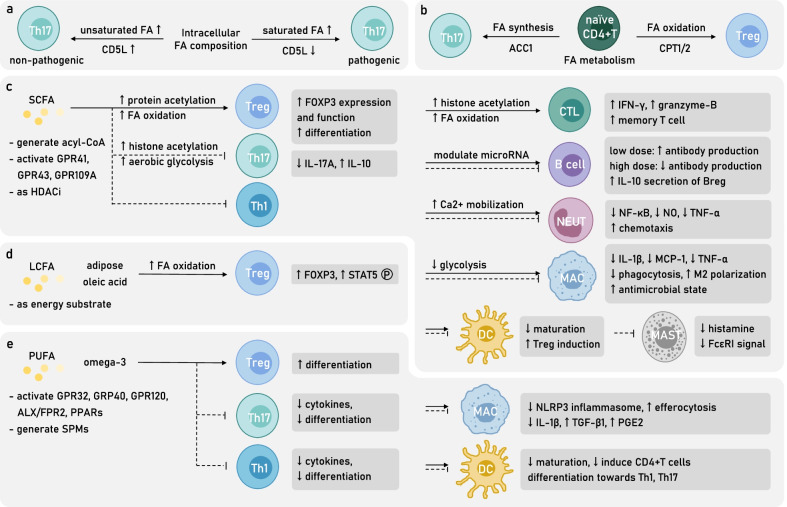
FAs and FA metabolism modulate immune cells’ differentiation and function in MS. **a** Intracellular FA composition is related to Th17 pathogenicity as the CD5L-prompted rise of unsaturated FA proportion facilitate the maintenance of a non-pathogenic state. **b** Intracellular FA metabolic pattern witnesses a significant difference among CD4^+^T helper cells which facilitates their diverged differentiation. FA synthesis (FAS) initiated by ACC1 favors the differentiation towards pathogenic Th17, while FA metabolism indispensable of CPT1/2 induces the differentiation towards protective Treg. Extracellular SCFA, LCFA, and PUFA as metabokines manifest broad immunomodulatory property (**c**–**e**). **c** SCFAs, by manipulating protein acetylation and inner metabolic state, enhance Treg differentiation, and transform various innate immune cells into a more protective state and, meanwhile, reduce the number and function of Th17 and Th1 cells. Interestingly, SCFAs regulate B lymphocytes function in a dose-dependent manner. **d** Adipose-resident oleic acid, as a kind of LCFA, increases FA oxidation (FAO) of Treg, leading to a boost of its regulative function. **e** PUFAs, especially omega-3 DHA, induce the CD4^+^T naïve cells differentiation into Treg while dampening proinflammatory cytokine secretion of Th17, Th1 cells. Omega-3 PUFAs also exhibit modulatory role for innate immune cells, favoring the resolution of inflammation. *MAC* macrophage, *MAST* mast cell, *NEUT* neutrophil

FA metabolism is indispensable for immune cells function. For example, the blockage of either FA oxidation (FAO) or synthesis (FAS) in dendritic cells (DC) inhibits their activation, though does not affect their survival [90]. Interestingly, FAs metabolic patterns participate in the modulation of T lymphocyte subtype differentiation. It is broadly acknowledged that MS pathogenicity emphasizes an abnormal activation of autoimmune Th1, Th17 cells, yet an inhibition of Treg cells, suggesting an underestimated FAs metabolic disorder among autoimmune T lymphocytes. Meanwhile, an adequate intervention of FAs metabolism may present immunomodulatory capacity. In particular, the survival and activation of Th17 cells depend on aerobic glycolysis and FAS, somehow that of Treg cells tend to rely on FAO and oxidative phosphorylation [91, 92]. Considering the dynamic process of FAs oxidation and synthesis, a restriction to rate-limiting enzymes on either side would regulate the balance of the Th17–Treg axis. For example, ACC1 and FASN blockage or knockout inhibits Th17 differentiation while promotes Treg differentiation [93]; on the contrary, CPT1 blockage inhibits the differentiation of Treg cells [91, 92]. The adoptive transfer model of experimental autoimmune encephalitis (EAE) built on FASN inhibitor-treated Th17 cells significantly reduces CNS Th17 infiltration and disease severity [93]. Similarly, ACC1 blockage or inhibitor-treated MOG_35–55_ EAE features a compromised Th17 differentiation and proliferation ability, as well as an alleviation of symptoms [94]. Furthermore, by longitudinally analyzing the CD45.2^+^ ACC1-knockout CD8^+^ cytotoxic T lymphocytes (CTLs) transferred to CD45.1^+^ recipient mice, ACC1 deficiency is proved to hamper CTLs proliferation and activation, and upon Listeria monocytogenes OVA (LmOVA) infection, the survival and accumulation of antigen-specific CTLs, whereas in vitro supplementation of FAs can restore CTL stable survival and the potential of blastogenesis, suggesting a crucial role of de novo FAS for CTL as well [95]. The above evidence highlights the supportive role of FAS in MS pathogenic T lymphocyte subtypes, while a preference for FAO in T lymphocytes reflects the acquisition of immunoregulative capacity (Fig. 2b).

However, although Treg cells require an external supply of FAs to support oxidative phosphorylation, a lack of extracellular FAs or a decrease of surface fatty acid uptake receptor FABP5 would cause damage to mitochondria, leading to a release of mtDNA into cytoplasm which induces the activation of type I IFN signaling pathway, related surface marker expression, and IL-10 secretion, promoting immunomodulatory function of Treg cells instead [96]. Therefore, blocking FABP4/5 would impede the transportation of FAs for successive oxidation, yet it still reduces the severity of EAE symptoms due to the compensation of Treg stimulation [97].

Furthermore, it is worth noting that the pathogenicity of Th17 cells can be regulated by intracellular SFA/PUFA ratio with the help of CD5L. CD5L, as a soluble protein, is reported to bind cytosolic FASN in adipocytes to favor lipolysis [98]. In Th17 cells, CD5L expression increases intracellular PUFA, while reduces SFA and MUFA levels, which would then affect RORγt ligand availability and its binding to *Il17a* and *Il23r* enhancer, thus facilitating the maintenance of a non-pathogenic Th17 state [99]. In short, SFA/PUFA ratio serves as a metabolic switch to regulate Th17 cell functional state, highlighting that the balance of FAs composition, or namely, the desaturation degree of intracellular FAs for particular T lymphocytes subtype would decide their innate function (Figs. 2a, 4).

### SCFAs and CD4^+^T lymphocyte subtype prevalence

Recently, SCFAs as extracellular “metabokines” have demonstrated potential treatment value due to their broad immunomodulatory effects. Of note, as mediators of intestinal homeostasis contributing to peripheral immune balance, SCFAs are particularly important targets in restoring MS gut–brain axis perturbation [50]. For example, MS intestinal microbiota composition witnesses a significant reduction of SCFAs-producing bacteria compared to healthy controls [100], which is sufficient to deliver an EAE susceptibility [101, 102], accordingly, high-fiber diet or direct SCFAs supplementation, succeeds in dampening disease activity [54].

At the cellular level, valerate not only inhibits Th17 cells of IL-17A secretion as an HDAC inhibitor but also increases their IL-10 secretion by boosting aerobic glycolysis and producing additional acetyl-CoA to assist histone acetylation [103]. Butyrate and propionate facilitate the extrathymic differentiation of Treg cells by promoting the acetylation of *Foxp3* promotor locus and FOXP3 protein, to enhance FOXP3 transcription and protein stability, as well as its TF function [77]. Apart from acting as an HDAC inhibitor, butyrate can be transformed into butyryl-CoA with the help of acyl-CoA synthetase short-chain family member 2 (ACSS2), to antagonize the inhibitory effect of malonyl-CoA on CPT, thus by upregulating CPT1A activity promotes FAO, and facilitates iTreg differentiation [104] (Fig. 2c).

SCFAs supplementation in EAE animal models has exhibited therapeutic effects (Table 2, Fig. 4). Dietary intake-related, gut microbiota-derived SCFAs inhibit JNK1/p38 and result in a long-lasting increase of intestinal Treg cells proliferation in a GPR43-dependent manner [105] while causing a reduction of Th1 cells level [106], proved to alleviate CNS axonal damage and relieve symptoms of EAE [54]. Methyl acetate administration alleviates EAE symptoms, CNS Th1, Th17 cells infiltration and demyelination by enhancing splenic chemokines expression that arrests Th1 cells in the peripheral [107].

**Table 2:** Summary of attempts exploring therapeutic effects of SCFA, MCFA and LCFA supplementation in MS animal models and patients

Study	Subjects	FA	Dosage and time	Main results
Zhang et al. [180]	Male Lewis rats MBP_68–84_ EAE	SCFA (valproic acid)	250 or 500 mg/kg per day for 19 days (preventive), 500 mg/kg per day for 12 days (therapeutic)	Preventive and therapeutic valproic acid treatment reduces EAE severity and CNS lesions by controlling CD4^+^T cells counts, inhibiting spinal cord inflammatory cytokines production while favoring a Th2 and Treg cytokine profile
Lv et al. [168]	Female C57BL/6 mice MOG_35–55_ EAE	SCFA (valproic acid)	10–300 mg/kg per day until the end of study	Valproic acid supplementation attenuates EAE clinical symptoms, T lymphocytes peripheral population and CNS infiltration by inhibiting T cells proliferation, while inducing caspase-dependent apoptosis. In vitro experiments of PBMC from HC and MS patients support the regulatory role valproic acid in T lymphocytes
Pazhoohan et al. [169]	Female Wistar rats Focal cortical EAE	SCFA (valproic acid)	300 mg/kg per day for 4 days (therapeutic) or 8 days (preventive)	Preventive valproic acid supplementation in a focal EAE model reduces severity, while a therapeutic treatment after EAE induction accelerates recovery. Overall, the valproic acid treatment enhances remyelination process measured by the ratio of myelin sheath thickness to axon diameter, with an increased recruitment of neural stem cells and oligodendrocyte progenitors to the lesion area
Haghikia et al. [54]	Female C57BL/6 mice MOG_35–55_ EAE	SCFA (propionic acid), MCFA (lauric acid)	200 µl (150 mM) propionic acid per day, or diet containing 4.2% lauric acid	SCFA propionic acid-rich diet promotes intestinal Treg polarization and endows Treg a more protective phenotype by inhibiting p38/MAPK, ameliorating EAE clinical score and CNS autoimmunity. On the opposite, a diet rich in MCFAs, particularly lauric acid, induces the differentiation and proliferation, as well as the CNS migration of intestinal Th1, Th17 cells by activating p38/MAPK. Detrimental effects of MCFA on intestinal CD4^+^T cells can be transferred through feces gavage to GF recipient mice
Liu et al. [158]	Male Lewis rats MBP EAE	SCFA (valproic acid)	250 or 500 mg/kg per day for 13 days	Valproic acid gavage alleviates EAE clinical scores, attenuates the local inflammation of optic nerves, by reducing pro-inflammatory cytokines expression and NF-κB pathway activity. Ultimately, valproic acid inhibits local microgliosis and caspase-dependent RGC apoptosis
Chevalier et al. [173]	Female C57BL/6 mice MOG_35–55_ EAE	SCFA (acetic acid)	4 g/kg per day until the end of experiment in the form of glyceryl triacetate	Acetate supplementation prevents EAE symptom onset, presumably by providing materials for FAS, rescues the loss of spinal cord ethanolamine and choline glycerophospholipid, as well as the phosphatidylserine, thus preserving myelin structural characteristics. Moreover, acetate supplementation reduces cPLA_2_ level which would contribute to downwards proinflammatory lipid signaling
Mizuno et al. [106]	C57BL/6 mice MOG_35–55_ EAE	SCFA (acetic acid, butyric acid, propionic acid)	200 mM in drinking water	High fiber diet or oral administration of SCFAs including acetic acid, butyric acid and propionate acid ameliorate EAE symptoms by upregulating Treg population. Ex vivo T cells from EAE after SCFAs supplementation reduces inflammatory cytokines production upon stimulation
Luu et al. [103]	C57BL/6 mice MOG_35–55_ EAE	SCFA (valeric acid)	150 mM in drinking water	Valeric acid, by promoting histone acetylation and mTOR activation, is able to enhance lymphocyte glucose oxidation. Valeric acid supplementation in EAE supports Breg immunomodulatory property while dampens Th17 function, succeeds in reversing an increase of EAE susceptibility prompted by detrimental gut bacteria colonization
Chen et al. [170]	Male C57BL/6 mice Cuprizone-induced demyelination	SCFA (butyric acid)	200 mM in drinking water	In vivo butyric acid supplementation ameliorates cuprizone-inducing demyelination in a microglia-independent way. In vitro assays utilizing organotypic cerebellar slice cultures further verify the ability of butyric acid both in attenuating acute demyelination and enhancing afterwards remyelination process, by acting directly to promote OPC differentiation as HDACi
Duscha et al. [21]	RRMS, SPMS, PPMS patients	SCFA (propionic acid)	500 mg twice daily	A reduced amount of propionic acid is found in MS patients’ serum and stool compared to HC. Two weeks of propionic acid supplementation as add-on therapy in MS patients rescues the imbalance of CD4^+^T cells differentiation and enhances Treg function by restoring mitochondrial respiration. Three years of supplementation significantly reduces MS ARR, disease progression and brain atrophy. The protective effect of propionic acid supplementation can be delivered by patients’ gut microbiota to an artificial murine gut culture system
Haase et al. [108]	C57BL/6 mice MOG_35–55_ EAE	SCFA (propionic acid)	150 mM in drinking water	Propionic acid has a lower concentration in obese MS patients compared to non-obese MS patients. Propionic acid supplementation reverses the detrimental effects of lauric acid-enriched diet in EAE, involving the rise of clinical scores, CNS infiltration of macrophages and T lymphocytes. Mechanistically, in vitro studies show that propionic acid by reducing p38–MAPK phosphorylation, restores Treg–Th17 axis homeostasis both in animal models and in patients
Pompura et al. [111]	RRMS patients	LCFA (oleic acid)	(in vitro)	Oleic acid, as the most prevalent adipose FA, has a comparative low concentration in MS adipose tissue. Oleic acid by enhancing FAO, promotes Treg expression of FOXP4 and pSTAT5, thus inducing a more immunomodulatory phenotype of Treg cells. MS PBMC or adipose-resident Treg cells are transcriptionally similar to ARA-treated HC PBMC, instead of oleic acid-treated ones which resemble HC Treg cells. In vitro studies verify the therapeutic potential of oleic acid treatment in rescuing MS Treg immunosuppressive effects
Xie et al. [107]	Male C57BL/6 mice MOG_35–55_ EAE	SCFA (acetic acid)	10, 30, or 100 mg/kg per day until the end of study in the form of methyl acetate	Methyl acetate reduces EAE severity, CNS T lymphocytes infiltration and demyelination by upregulating splenic expression of chemokines which attracts Th1 cells in the peripheral and blocks their central recruitment. Methyl acetate also attenuates intestinal inflammation by reducing local Th1, Th17 cells counts
Zhu et al. [174]	Male C57BL/6 mice Cuprizone-induced demyelination	SCFA (valproic acid)	150 mg/kg per day	Valproic acid gavage mitigates cuprizone-induced myelin structure loss and anxiety-like behavior, by promoting hippocampal cholesterol biosynthesis

*ARR* annual relapse rate, *CNS* central nervous system, *cPLA*_*2*_ cytosolic phospholipase A2, *EAE* experimental autoimmune encephalomyelitis, *FAO* fatty acid oxidation, *FAS* fatty acid synthesis, *GF* germ-free, *HC* healthy control, *HDACi* histone deacetylase inhibitor, *LCFA* long-chain fatty acid, *MBP* myelin basic protein, *MCFA* medium-chain fatty acid, *MOG* myelin oligodendrocyte glycoprotein, *MS* multiple sclerosis, *OPC* oligodendrocyte precursor cell, *PBMC* peripheral blood mononuclear cell, *PPMS* primary progressive multiple sclerosis, *RRMS* relapsing–remitting multiple sclerosis, *SCFA* short-chain fatty acid, *SPMS* secondary progressive multiple sclerosis

Among all SCFAs, the protective effect of propionic acid is shown to be the most significant, which has been validated among MS patients [106] (Table 2). A significantly reduced propionic acid level is found in serum and feces of MS patients, including those newly diagnosed ones [21]. Meanwhile, a 2-week supplementation of propionic acid (1000 mg/day) in MS patients is sufficient to rescue the impaired Treg proliferation and control the overactivated Th1, Th17 level [21]. In addition, a 3-year supplementation of propionate as add-on therapy significantly reduces the annual relapse rate (ARR), slows down disease progression, ameliorates symptoms, and reverses brain atrophy [21]. Mechanistically, propionate serves to regulate T lymphocyte subtype balance, restores mitochondrial morphology and function by regulating a series of mitochondrion-related genes particularly carnitine* O*-octanoyl transferase (*CROT*), which is responsible for the translocation of acyl-CoA from peroxisomes to mitochondria for the subsequent FA oxidation [21]. Similar result has been achieved among obese MS patients, who accommodate an even lower feces propionic acid concentration compared with non-obese MS patients [108]. Experiments conducted in EAE animals verify a protective role of propionic acid supplementation which reverses the high fat diet (HFD)-induced aggravation of demyelination and clinical score, as well as the CNS infiltration of macrophages and T lymphocytes [108]. Further results indicate that propionic acid by rescuing the HFD-induced over-phosphorylation of p38–MAPK, restores the balance of Th17–Treg axis both in animal models and in obese MS patients [108].

In fact, indole–propionic acid derivatives have been developed as the sphingosine-1-phosphate (S1P) receptor agonist [109]. An optimized selective S1P_1_ receptor agonist 9f, which avoids the vascular side effect upon S1P_3_ activation, has shown equivalent efficacy of reducing peripheral lymphocytes and EAE clinical score compared with fingolimod [110].

### MCFAs, LCFAs and CD4^+^T differentiation

Unlike SCFAs, MCFAs are suggested to mediate destructive events in MS pathogenesis (Table 2, Fig. 4). MCFA, such as caproic acid, is reported to have a high serum concentration in MS patients compared to healthy controls, and is positively correlated with Th1 subset ratio [57]. In vivo studies show that MCFAs, including caproic acid, caprylic acid, capric acid and lauric acid-enriched diet exacerbate EAE symptoms by promoting the intestinal differentiation and proliferation of Th1 and Th17, as well as their migration into the peripheral blood and CNS through p38–MAPK activation [54]. Interestingly, the detrimental effects of MCFAs to intestinal resident CD4^+^T lymphocyte phenotype can be delivered by feces transfer to recipient germ-free (GF) mice, suggesting that MCFAs by targeting gut–brain axis mediate lifestyle-related MS susceptibility [54].

However, adipose-resident LCFA oleic acid bears a protective role and is observed to be decreased in MS patients [111]. Further investigation showed that adipose oleic acid indeed improves the oxidative phosphorylation level of Treg cells by supporting FA oxidation, thus increasing their FOXP3 expression and STAT5 phosphorylation, boosting Treg function [111] (Fig. 2d). This finding points out a tissue-resident modulatory role of LCFAs, highlighting that the storage of FAs within adipose tissue somehow would influence overall immune response (Table 2).

### PUFAs and CD4^+^T differentiation

As has been discussed before, PUFAs are divided into two categories, namely, omega-3 and omega-6 PUFAs. While the high MS incidence of the western world suggests a potential detrimental effect of omega-6 PUFAs-enriched western diets [112], a diet dominated by omega-3 PUFAs, especially of fish oils-enriched, has been found to reduce the risk of developing MS [113], to improve MS clinical outcome [26, 114], and to modulate MS-related inflammatory cytokines [22, 26]. As we focus on the role of PUFAs in MS susceptibility and their therapeutic potential, a brief recapitulation of their biological nature in MS-related pathological process is provided.

Omega-3 PUFAs exhibit immunomodulatory effects and are known to ameliorate EAE (Fig. 4). For example, DHA generates downstream SPMs including Resolvin D1/2 and Maresin1 which inhibit CTL activation and proinflammatory cytokines secretion of Th1, Th17 cells from peripheral blood mononuclear cells (PBMC), whereas does not affect their proliferation [115]. Meanwhile, DHA, EPA by directly targeting GPR32, GPR120 and ALX/FPR2, inhibit CD3^+^T cells activation [116], Th1, Th17 differentiation while inducing differentiation towards Foxp3^+^iTreg [115]. Furthermore, a diet rich in DHA, EPA for EAE mice alleviates EAE score, increases the expression of PPAR-α, γ, β/δ in CNS CD4^+^T cells and, meanwhile, reduces the CNS infiltration of IFN-γ and IL-17-secreting CD4^+^T cells [117, 118].

However, omega-6 PUFAs polarize CD4^+^T cells towards a more pathogenic phenotype. LA treatment on CD4^+^T cells ex vivo promotes the exacerbating role of NaCl which induces Th17 differentiation and proliferation, as well as their secretion of pathogenic cytokines [119] (Fig. 2e).

### FAs and CTL function

Besides their role in CD4^+^T lymphocytes, butyrate and propionate act as HDAC inhibitors to promote the secretion of IFN-γ and granzyme B in CTL and Tc17 cells [120]. In a local environment lacking glucose, acetate absorbed by CTL is transformed into acetyl-CoA with the help of ACSS2, which would thereby promote histone acetylation and the activation of related genes, boosting CTL function [121]. SCFA, especially butyrate, by acting on GPR41/43, promotes glycolysis and FA oxidation to support oxidative phosphorylation in CTLs, prompting the formation of memory T cells and the preservation of immune memory [122] (Fig. 2c).

### FAs modulate B lymphocyte function

Butyrate and propionate, but not acetate, as HDAC inhibitors affect downstream miRNA, and interfere with B lymphocytes antibody production in a dose-dependent manner. Low dose generates miRNAs boosting antibody class switch and recombination, yielding more antibodies, while high dose produces a large number of miRNAs targeting *Aicda* and *Prdm1*, thus inhibiting class switch and the generation of systemic antigen-specific antibodies [78]. SCFA, by activating GPR43 which is highly expressed among marginal zone B cells, would putatively inhibit antibody production against non-T cell-dependent antigens [123]. In addition, valerate can induce IL-10 production of Breg cells so as to relieve EAE symptoms and infiltration of inflammatory cells [103] (Figs. 2c, 4).

The interactions of different etiological co-factors such as infections and FAs may be mediated by B cells. A recent large-scale cohort study published supports a concrete correlation between MS incidence and Epstein–Barr virus (EBV) infection [124], reinforcing the EBV-prompted etiology hypothesis [125–127]. In brief, the infection would putatively lead to abnormal accumulation of autoimmune EBV-infected B cells majorly of latent phase, triggering CNS lymphocytes colonization and chronic inflammation [127]. Specifically, SCFAs, such as butyrate induces [128], whereas MCFAs inhibit [129] EBV-infected B cells to enter the lytic phase. However, there are inconsistent results [130, 131]. These findings suggest the potential interactions of FAs and EBV infection through the virus lytic procedure in the development of the disease, while how FAs are engaged remains obscure [132], hence expecting more investigations to clarify this relationship.

### FAs regulate innate immune cells

More have been found regarding their role in innate immune cells. As for neutrophils, SCFAs binding GPR43 and coupling Gi/o and Gq results in intracellular Ca^2+^ mobilization and induces neutrophils chemotaxis towards their direction [133, 134]. SCFAs, including acetate, propionate, and butyrate act as HDAC inhibitors to inhibit NF-κB and the secretion of proinflammatory factors including NO, TNF-α, etc. in neutrophils [133].

For macrophages, butyrate as HDAC inhibitor increases H3K9 acetylation and STAT6 expression to induce macrophage M2 polarization [135]. By inhibiting HDAC, glycolysis and mTOR pathway, while promoting autophagy and the expression of calprotectin, butyrate can induce macrophages functional differentiation into an antimicrobial state [136]. Formate and valerate in their physical concentration can reduce the secretion of IL-1β, CCL2, and TNF-α of monocyte-like THP1 cells in inflammatory conditions, at the same time inhibit their phagocytosis function, among which the effect of formate is GPR41/43-dependent [137] (Fig. 2c). Omega-3 DHA by activating PPARγ promotes macrophages’ efferocytosis so as to participate in the resolution of inflammation [138]. In addition, DHA, EPA, and ALA act through GPR120/40 and their scaffold protein β-arrestin-2 to inhibit induced NLRP3 inflammasome activation and IL-1β secretion of macrophages [139]. DPA can also inhibit the transcriptional level of LPS-induced proinflammatory cytokines secretion in macrophage-like RAW264.7, independent of their transformation to DHA [140]. SFA activates TLR-related proinflammatory signal pathways in macrophages and neutrophils; however, DHA has the opposite effects [70]. Feeding EAE mice with omega-6 γ-linoleic acid-rich plants ameliorate disease severity and reduce relapses, by raising splenocyte membrane-detected ARA precursors and ARA level, which would generate downstream immunomodulatory factors PGE1 and PGE2, thus increasing the expression of TGF-β1 and PGE2 in splenic monocytes [141] (Fig. 2e).

For DCs, butyrate as HDAC inhibitor and GPR109A activator inhibits PBMC-derived LPS-induced DC maturation, meanwhile, boosting DC ability to induce CD4^+^T cells to differentiate into IL-10 secreting Treg cells [77, 142] (Fig. 2c). Similarly, DHA also inhibits LPS-induced DC maturation [140]. DHA-treated DCs, by activating GPR120, downregulate co-stimulatory molecules [143], thus failing to induce antigen-specific T cells to proliferate and differentiate into Th1 and Th17 cells, instead increasing their expression of TGF-β and FOXP3 [144] (Fig. 2e).

There is even regulation towards mast cells. Butyrate and propionate as HDAC inhibitors induce downregulation of FcεRI downstream tyrosine kinase BTK, SYK, LAT in mast cells, inhibiting allergen-prompted histamine release [139] (Fig. 2c).

### Downstream bio-mediators and chronic inflammation

MS experiences a state of chronic inflammation, which usually accompanies a dysregulation of lipid mediators, namely, classical eicosanoids and SPMs [88]. Evidence has accumulated that MS peripheral blood witnesses a higher level of classical eicosanoids and SPMs, moreover, their increase is associated with disease progression [145, 146]. Due to a lack of investigations, inner pathological events have not been clarified yet. The fluctuation of biofluid mediators among MS patients might due to compensation effects, or on the contrary, serves as the cause that hinders the recovery of CNS lesions. However, what can be sure is that dietary intervention can adjust the systemic level of lipid mediators and helps in the resolution of inflammation. A randomized and crossover-controlled clinical trial conducted among healthy people has verified that dietary supplementation of omega-3 PUFA helps in generating more SPMs in response to inflammation [147]. Feeding omega-3 PUFAs in newborn mice for 2 months increases their hippocampus PUFA contents and, meanwhile, generates more anti-inflammatory, less proinflammatory oxylipins and cytokines upon LPS challenge [148].

## FAs with multifaceted roles in MS CNS pathogenesis

MS is not only an autoimmune disease, but also indeed a neurodegenerative disease. Therefore, the contributors to late-stage irreversible CNS damage cannot be ignored, such as long-term BBB damage, oxidative stress, remyelination failure, axonal energy failure, etc. [5, 89]. Evidence is accumulating that FAs and FA metabolism are crucial mediator of CNS homeostasis, exhibiting multifaceted role in MS-related CNS pathogenesis.

### FAs prevent blood–brain barrier leakage

**Fig. 3:**
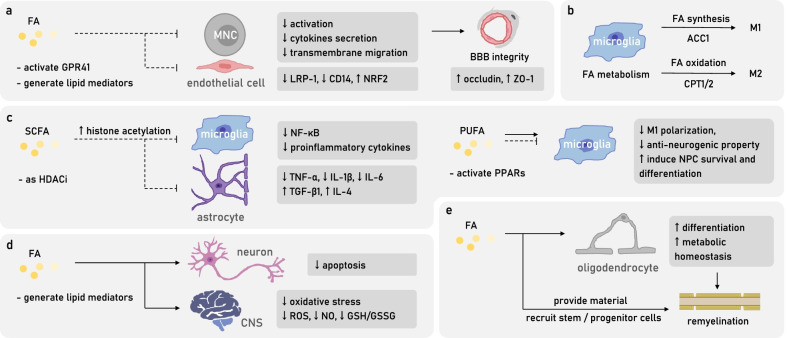
FAs with multifaceted roles in MS CNS pathogenesis. **a** FAs, by inhibiting the inflammation-induced MNCs activation and migration, as well as by alleviating oxidative stress of endothelial cells, help to preserve BBB integrity during the pathogenic state. **b** FA metabolism modulates the polarization of microglia, which are critical contributors of MS late-stage CNS lesions. **c** FAs regulate CNS resident glia by introducing them to an anti-inflammatory and pro-neurogenic state. **d** FAs reduce neuron apoptosis and CNS oxidative stress. **e** FAs promote remyelination by providing raw materials, recruiting stem cells and progenitor cells, and by favoring oligodendrocytes differentiation and metabolic homeostasis. *BBB* blood–brain barrier, *CNS* central nervous system, *MNC* mononuclear cell

MS patients have a compromised BBB which allows the entry of immune cells into the CNS, while FAs are known to preserve BBB integrity. FA downstream lipid mediators LXA4, LXB4, RvD1, and PD1 can reduce the activation and the secretion of cytokines in MS-derived mononuclear cells, inhibit inflammation-induced BBB breakdown, as well as the transmembrane migration of mononuclear cells [145]. SCFAs as gut microbiota-derived molecules are indeed indispensable in maintaining BBB integrity. Fecal transplantation of SCFAs-producing microbiota or direct gavage of butyrate can reverse the BBB leakage of GF mice, restore tight junction protein expressions, such as occludin and ZO-1 [149, 150]. During in vitro challenge of LPS-induced oxidative stress, SCFA propionate acts on GPR41 of human brain endothelial cells to inhibit the expression of efflux transporter protein LRP-1, downregulate CD14 transcripts, and induce cytoplasm translocation of NRF2 to protect BBB [151] (Fig. 3a).

### FAs modulate CNS resident glia

In the late stage of MS disease, the principal contribution to CNS autoimmune damages experiences a shift from adaptive immunity to innate immunity, reflected by the presence of inflammatory macrophages and microglia in subcortical and smoldering lesions, resulting in demyelination, axonal injuries, and continuous expansion of lesions [152]. Accumulating evidence has indicated that the inherent metabolic pattern of macrophage lineages would affect M1/M2 phenotype polarization, thus contributing to the local persistence of inflammation [153] (Fig. 3b). For example, the proinflammatory M1 cells rely on the pentose phosphate pathway and FA synthesis, whereas the anti-inflammatory M2 cells have active FA oxidation and oxidative phosphorylation [152]. CPT1 inhibitor which blocks the entry of FA into mitochondria and the successive oxidation can directly restrain the M2 polarization of macrophages and microglia [154]. Furthermore, FAs, especially SCFAs, as “metabokines” easily cross the BBB and practice modulatory roles among CNS glia (Fig. 3c). Acetate supplementation in vivo increases CNS overall acetyl-CoA and histone acetylation level, reducing LPS-induced glia activation and IL-1β secretion [155]. Acetate treatment in microglia in vitro can reverse the LPS-induced low acetylation level of H3K9, manipulate PTM of signaling pathway proteins, including phosphorylation level of p38, MAPK, JNK, etc., and acetylation level of lysin310, to modulate their inflammatory cytokines production [155]. Butyrate as an HDAC inhibitor also reduces LPS-induced microglia activation and CNS inflammatory cytokines production [156]. VPA ameliorates EAE early symptoms [157], mitigates optic neuritis local inflammation and microglia proliferation, inhibits the expression of cytokines including INF-γ, TNF-α, IL-1β, IL-17, iNOS as well as downregulates NF-κB pathway, and CD11b as the marker of macrophages and microglia [158]. DHA, by activating PPAR-γ and inhibiting p38/MAPK phosphorylation, restrains the LPS-induced microglia M1 polarization and reverses their anti-neurogenic function, instead renders them a supportive role for neural progenitor cell (NPC) survival and differentiation [159]. Apart from microglia, for LPS-induced astrocyte inflammation in vitro, acetate by inducing H3K9 acetylation, can affect MAPK and NF-κB signaling pathway, so as to inhibit TNF-α, IL-1β, IL-6 while promoting TGF-β1 and IL-4 production [160].

### FAs and CNS oxidative stress, apoptosis

**Fig. 4:**
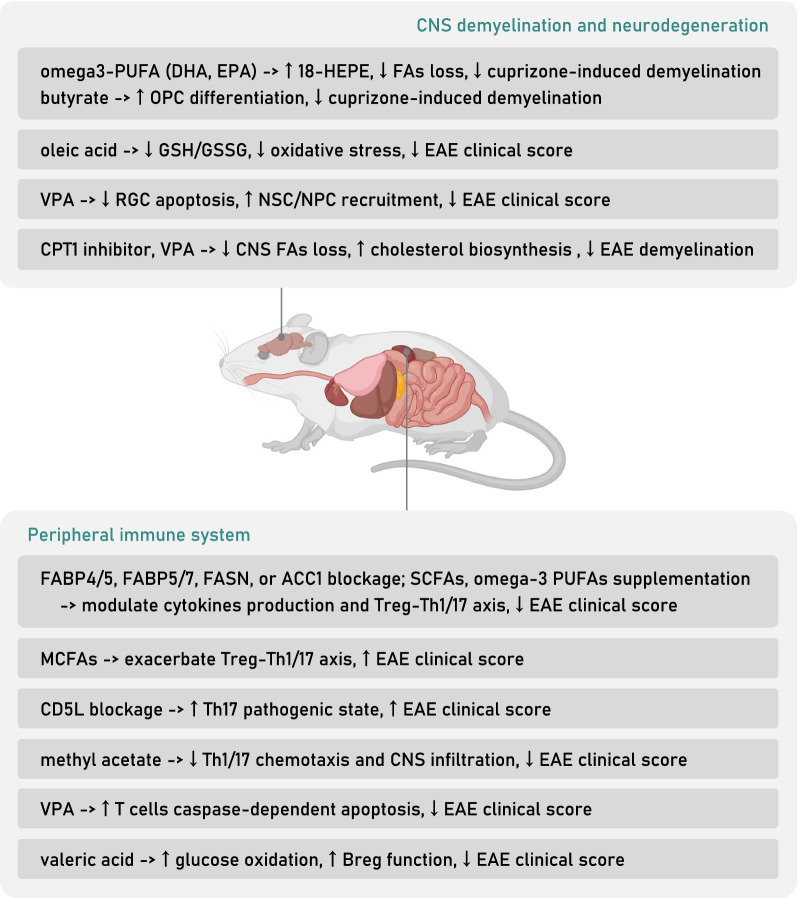
Attempts of targeting FAs and FA metabolism in MS animal models. A blockage of FAs chaperones or critical FAS enzymes, including fatty acid binding protein 4/5 (FABP4/5), FABP5/7, fatty acid synthase (FASN) and ACC1, by inducing FAO in peripheral immune system, manages to restore the proper balance of Treg–Th1/17 axis, mitigating EAE severity. SCFAs and omega-3 PUFAs including DHA or EPA treatments are also able to reduce EAE clinical score by manipulating CD4^+^T cells differentiation and subtype function. The blockage of CD5L, an inhibitor of FASN function, serves to increase SFA/PUFA ratio in Th17 cells, favoring a more pathogenic phenotype, therefore, aggravating EAE symptoms. Methyl acetate treatment by reducing Th1, Th17 chemotaxis and CNS infiltration mitigates EAE. Valproic acid (VPA) supplementation alleviates EAE by promoting T cells apoptosis. Valeric acid supplementation boosts Breg function which leads to EAE remission. In CNS pathogenic state, omega-3 PUFAs and butyrate either by increasing anti-inflammatory bio-mediators or by directly supporting oligodendrocyte precursor cell (OPC) differentiation, alleviate cuprizone-induced demyelination. Oleic acid supplementation relieves EAE symptoms by reducing oxidative stress as the decrease of GSH/GSSG ratio. VPA mitigates EAE by reducing retinal ganglion cells (RGC) apoptosis and by recruiting neural stem or progenitor cells (NSC, NPC). Critical FAO enzyme CPT1 inhibitor and VPA administration alleviate EAE demyelination by reducing FA loss while boosting FA and cholesterol biosynthesis. *EAE* experimental autoimmune encephalomyelitis, *PUFA* poly-unsaturated fatty acid, *SFA* saturated fatty acid

FAs have also established a protective role in CNS degeneration featuring oxidative stress and neuronal apoptosis (Figs. 3d, 4).

The intense oxidative stress of local lesions is one of the major late-stage pathological features of MS, generating by resident cells including microglia, astrocytes, and neutrophils, or as downstream products of omega-3 AA metabolism. The high oxygen consumption rate, low level and activity of antioxidant enzymes, and the high level of easily oxidized PUFAs, all render CNS vulnerable to oxidative damage [161]. PUFAs, such as ARA, DHA can be easily oxidized by ROS and NO due to their fragile carbon double-bonds, which thereby, causes damage to the cell membrane and myelin sheath, yielding byproducts of lipid peroxidation including 4-hydroxy-2-nonenal (HNE) to mediate long-distance protein and DNA damage [161]. Meanwhile, certain FAs are known to possess antioxidant effect. Formate can reduce fMLP-induced respiratory burst of myeloid neutrophils HL-60 so as to decrease ROS production [137]. LA can competitively inhibit the proinflammatory effects of ARA, producing PGE1/2 to inhibit the production of proinflammatory cytokines [45]. Oleic acid supplementation in EAE significantly reduces the GSH/GSSG ratio, TNF-α, NF-κB p65, NO level, etc. in brain tissue, spinal cord, and peripheral blood, demonstrating an antioxidant effect [162] (Fig. 4).

Furthermore, some FAs are beginning to demonstrate some anti-apoptotic properties (Fig. 4). VPA administration inhibits the apoptosis and related signaling pathway of EAE retinal ganglion cells (RGCs) [158] (Fig. 4). Dietary supplementation of a balanced mixture of FAs, namely, neuro-FAG in EAE mice reduces the transcription and expression of various proinflammatory cytokines, while does not affect the macrophage secretion of RGC-trophic factors oncomodulin (OCM), indeed reducing the loss of RGCs and postponing EAE symptom onset [163].

### FAs and remyelination

CNS remyelination process requires FAs as raw materials (Fig. 3e). Oleic acids as the major composing FAs of the myelin sheath can be synthesized and secreted by astrocytes to promote oligodendrocyte remyelination [45]. Inducing demyelination with cuprizone in fat-1 mice results in an increase of remyelination degree during recovery, accompanied by a rise of CNS EPA and downstream bioactive 18-HEPE compared to WT mice, indicating an assisting role of omega-3 PUFAs in the process of remyelination [164]. Accordingly, feeding salmon fillet diet rich in omega-3 PUFAs to cuprizone-induced demyelination models results in an alleviation of MRI lesions and corpus callosum demyelination, as well as an increase of remyelination level; however, for cod liver oil diet also rich in omega-3 PUFAs, or soybean oil diet rich in omega-6 PUFAs, no such results are observed, highlighting the needs to specify effective components [165] (Fig. 4). Besides barely providing materials, omega-3 EPA is known to stimulate myelin gene expression, aiding oligodendrocyte survival in the spinal cord trauma [166]. A continuous supplementation of EPA for 21 days before the induction of cuprizone demyelination model can significantly prevent the loss of CNS cerebroside and other components in comparison to the control group [167] (Fig. 4).

There is also evidence implying a protective role of FAs towards remyelination by recruiting stem and progenitor cells (Figs. 3e, 4). For example, VPA promotes remyelination by enhancing the recruitment of neural stem cells and oligodendrocyte precursor cells (OPCs) to local lesions [168, 169]. Butyrate as an HDAC inhibitor significantly mitigates the cuprizone/lysolecithin LPC-induced demyelination by inducing OPCs differentiation and maturation [170].

Considering that many genetic lipid metabolic diseases are often accompanied by CNS damage, MS CNS lipid loss and demyelination may not only owe to immunological reasons but also have the participation of CNS lipid metabolic disorder. Previous understanding of MS demyelination process emphasizes the loss of oligodendrocytes and their regeneration failure. However, evidence from pathological analysis points out an existence of approximately 30–60% lesions with almost intact oligodendrocytes [171]. Meanwhile, CNS lesions of primary progressive MS (PPMS) patients exhibit a more significant reduction of lipid compared to protein [172]. To address the inequivalence, researchers have confirmed an extensive downregulation of FA metabolism-related genes through transcriptome sequencing, indicating an undeniable dysregulation of FA metabolism, which can be presumably ameliorated by dietary intervention [172]. For instance, acetate supplementation, by increasing acetyl-CoA level, replenishes CNS energy storage and FA synthesis, to partially reverse EAE-induced myelin loss [173]. VPA supplementation, on the other hand, mitigates the cuprizone-induced demyelination and the associated anxiety-like behavior by restoring hippocampal cholesterol biosynthesis which probably facilitates oligodendrocyte remyelination [174]. In addition, administration of CPT-1 inhibitor in EAE mitigates CNS demyelination, indicating a probable positive role of restraining FA oxidation [175] (Figs. 3e, 4).

## Conclusions and perspectives

Researches into the potential therapeutic effect of PUFAs have yielded promising results in EAE rodent models, though, inconsistent findings spring up from bench to bedside [26]. However, it is not time yet to be disappointed as the lack of standardized implementation continues to be a serious problem of existing clinical trials, including the discrepancy of doses, sources, and treatment duration [114, 176]. Moreover, most studies are still of small sample size, with incomplete control groups, and a lack of attention paid to the actual circulating PUFAs concentration after quantitative oral supplementation [8, 177]. All these reasons bring difficulty in evaluating the actual benefits of FA supplementation in MS patients. Considering the limitation of EAE rodent models, the investigation into therapeutic PUFAs is still waiting for more standardized attempts and should emphasize the value of clinical translation.

Although the therapeutic efficacy of PUFAs for MS patients is still not definitive, FAs dietary intake pattern is clearly related to MS/CIS incidence risk [46, 113, 178, 179], which provides a proof-of-concept for the significance of metabolic memory over autoimmune diseases. It is worth noting that due to the presence of an asymptomatic preclinical stage among MS patients [4], applying beneficial FAs during the time window might ultimately prevent the occurrence of disease.

Compared with the uncertainty regarding the therapeutic effect of PUFAs, the application value of SCFAs in MS treatment is of promising prospect. Recently, researchers have tried to introduce propionic acid as an add-on therapy to MS conventional DMT treatment, which significantly prevents disease progression, rebalances Th cells subtype and mitochondria metabolism, providing MS patients with milder and more economical treatment options [21].

**Fig. 5:**
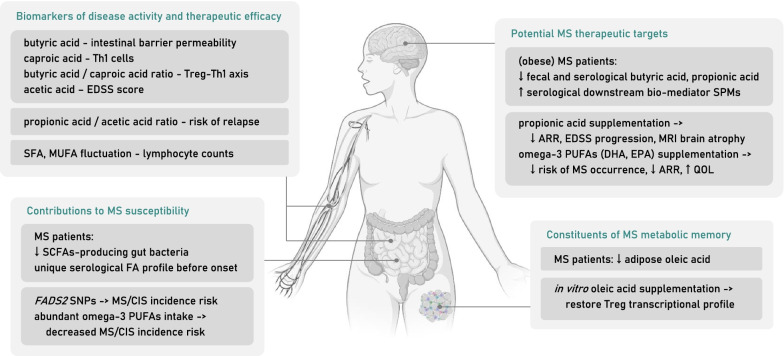
Proposed role of FA and FA metabolism in MS patients. First, FAs can serve as biomarkers of disease activity and therapeutic efficacy. Multiple FAs serological concentration can reflect disease activity, including intestinal barrier permeability, Treg–Th1 axis balance and EDSS score. A longitudinal cohort of pregnant MS patients indicates the predictive value of FAs ratio in determining risk of relapse. Moreover, after DMF treatment, drop of lymphocyte counts correlates the fluctuation of serological SFA and MUFA level. Second, FAs intake or metabolic state contributes to MS susceptibility. MS patients, long before onset, acquire a unique FA serological profile. Gut microbiome data indicates a preferentially decrease of SCFAs-producing bacteria in MS patients. Several FA metabolism-related enzyme single nucleotide polymorphisms (SNPs), and PUFAs intake patterns are related to MS incidence. Third, FAs are potential MS therapeutic targets. As FAs and related bio-mediators level are altered among MS patients, adequate supplementation helps to reduce MS incidence risk, annual relapse rate (ARR), clinical score, CNS pathology and quality of life (QOL). Fourth, FAs biological compositions are constituents of MS metabolic memory that would influence immune system. A significantly decreased level of adipose-resident oleic acid among MS patients leads to a pro-inflammatory transcriptional profile of Treg cells, which can be reversed by oleic acid supplementation. *CIS* clinically isolated syndrome, *EDSS* Expanded Disability Status Scale, *FADS* fatty acid desaturase

MS has dual pathological characteristics including an early-stage immune-mediated inflammatory response and late-stage secondary neurodegeneration. As have been illustrated, FAs as “metabokines” indeed harbor complex regulatory roles in intervening immune cells subtype differentiation and the balance of phenotype polarization; meanwhile, their critical role in CNS oxidative stress and remyelination cannot be under-appreciated. Taking advantage of research conducted among MS patients, a proposed relationship between FAs and MS is summarized in Fig. 5. As FAs are emerging as potential therapeutic targets, further in-depth exploration is still required to validate the reliability of clinical translation.

## Data Availability

Data sharing is not applicable to this article as no data sets were generated or analyzed during the current study.

## Abbreviations

ACC1: Acetyl-CoA carboxylase 1
ACSS2: Acyl-CoA synthetase short-chain family member 2
ALA: α-Linolenic acid
ARA: Arachidonic acid
ARE: AU-rich element
BBB: Blood–brain barrier
CIS: Clinically isolated syndrome
CNS: Central nervous system
CPT1/2: Carnitine palmitoyl-transferase 1/2
DC: Dendritic cell
DHA: Docosahexanoic acid
DMF: Dimethyl fumarate
DMT: Disease-modifying therapy
EAE: Experimental autoimmune encephalitis
ELOVL: Elongase of very long chain fatty acid
EPA: Eicosatetranoic acid
FA: Fatty acid
FABP: Fatty acid-binding protein
FADS: Fatty acid desaturase
FAO: Fatty acid oxidation
FAS: Fatty acid synthesis
FASN: Fatty acid synthase
GPCR: G-protein coupled receptor
HDAC: Histone deacetylase
HFD: High fat diet
HNE: 4-Hydroxy-2-nonenal
LmOVA: Listeria monocytogenes OVA
LNA: Linoleic acid
MCFA: Medium-chain fatty acid
MCP1: Monocyte chemotactic protein1
MS: Multiple sclerosis
MUFA: Mono-unsaturated fatty acid
NPC: Neural progenitor cell
OCM: Oncomodulin
OPC: Oligodendrocyte precursor cell
PBMC: Peripheral blood mononuclear cell
PPAR: Peroxisome proliferator-activated receptor
PPMS: Primary progressive multiple sclerosis
PTM: Post-translational modification
PUFA: Poly-unsaturated fatty acid
RBP: RNA-binding protein
RGC: Retinal ganglion cell
RRMS: Relapsing–remitting multiple sclerosis
S1P: Sphingosine-1-phosphate
SCFA: Short-chain fatty acid
SFA: Saturated fatty acid
SNP: Single nucleotide polymorphisms
SPM: Specialized pro-resolving mediator
SPMS: Secondary progressive multiple sclerosis
TLR: Toll-like receptor
VPA: Valproic acid
WT: Wild type
